# Survival rates and prognostic predictors of high grade brain stem gliomas in childhood: a systematic review and meta-analysis

**DOI:** 10.1007/s11060-017-2546-1

**Published:** 2017-07-05

**Authors:** Hadeel Hassan, Anne Pinches, Susan V. Picton, Robert S. Phillips

**Affiliations:** 1 Centre for Reviews and Dissemination, University of York, York, UK; 2 Department of Paediatric Haematology and Oncology, Leeds Teaching Hospitals NHS Trust, Leeds, UK

**Keywords:** Pediatrics, Brain stem glioma, DIPG, Prognostic, Survival and systematic review

## Abstract

Diagnosis of a pediatric high grade brain stem glioma is devastating with dismal outcomes. This systematic review and meta-analysis was undertaken to determine the survival rates and assess potential prognostic factors including selected interventions. Studies included involved pediatric participants with high grade brain stem gliomas diagnosed by magnetic resonance imaging or biopsy reporting overall survival rates. Meta-analysis was undertaken using a binomial random effects model. Sixty-five studies (2336 participants) were included. Meta-analysis showed 1 year overall survival (OS) of 41% (95% confidence interval (CI) 38–44%, I-sq 52%, 2083 participants), 2 year OS of 15.3% (95% confidence interval 12–20%, I-sq 73.1%, 1329 participants) and 3 year OS of 7.3% (95% confidence interval 5.2–10%, I-sq 26%, 584 participants). Meta-analyses of median overall survival results was not possible due to the lack of reported measures of variance. Subgroup analysis comparing date of study, classification of tumor, use of temozolomide, non-standard interventions or phase 1/2 versus other studies demonstrated no difference in survival outcomes. There was insufficient data to undertake subgroup meta-analysis of patient age, duration of symptoms, K27M histone mutations and AVCR1 mutations. Survival outcomes of high grade brain stem gliomas have remained very poor, and do not clearly vary according to classification, phase of study or use of different therapeutic interventions. Future studies should harmonize outcome and prognostic variable reporting to enable accurate meta-analysis and better exploration of prognosis.

**Electronic supplementary material:**

The online version of this article (doi:10.1007/s11060-017-2546-1) contains supplementary material, which is available to authorized users.

## Introduction

Brain stem gliomas (BSG) account for approximately 10–20% of all childhood CNS tumors. An estimated 350–400 pediatric cases (3–4 per 100,000 pediatric population) were diagnosed yearly in the United States of America (USA) from 2007 to 2011 [1] and 40 cases per year in the United Kingdom (UK) (approximately 3 per 100,000 pediatric population) from 1996 to 2005 [2]. Brainstem gliomas are not categorized according WHO classification of CNS tumors as with gliomas in other locations, rather they are grouped according to location and appearance using magnetic resonance (MRI) T1 and T2 weighted imaging [3, 4], although recently, there has been discussion of the need for histological diagnosis by biopsy and classification according to location [5]. They are classified broadly into two groups: low grade (or focal/exophytic) BSGs and diffuse intrinsic pontine gliomas (DIPG) including those confirmed histologically or those diagnosed using radiology alone. Diffuse tumors are typically infiltrating astrocytomas, which can be grade 2–4 depending on histopathological features and have a poorer outcomes when compared to focal BSGs [6–8]. These types of BSGs usually arise from the pons but can occur in other locations. When they arise from the pons they are called DIPGs and typically represent 80% of patients with BSG. Fractionated external beam radiotherapy daily for 6 weeks is standard therapy for pediatric high grade BSGs and chemotherapy with temozolomide is frequently offered. A Cochrane systematic review and meta-analyses by Hart et al. demonstrated a statistically significant increase in survival with the use of temozolmide in studies of adults with high grade gliomas (mortality hazard ratio 0.6, 95% CI 0.46–0.79). Although no previous meta-analyses have been completed on pediatric studies involving treatment with chemotherapy, the addition of chemotherapy does not clearly appear to improve survival in brain stem gliomas [9].

It has been hypothesized that certain features may be prognostic [6]. This includes duration of symptoms prior to diagnosis [7], age less than 3 years at diagnosis [10], histone H3 mutations [11], and ACVR1 mutations. Studies looking into the use of chemotherapy, immunotherapy, alternate radiotherapy including phase 1, 2 and 3 trials have not been able to demonstrate any significant improvement in outcome [9]. This is the first systematic review to estimate survival outcomes and assess proposed prognostic factors in pediatric high grade BSGs.

## Methods

This systematic review followed a prespecified protocol which was registered on PROSPERO [12] an international database of health and social care systematic reviews (PROSPERO 2013:CRD42013006592).

### Inclusion criteria

Designs of studies eligible included randomized-controlled trials (RCTs), quasi-RCTs and observational studies such as case-control, cohort studies including phase 1 and 2 trials. Only studies of pediatric participants diagnosed with high grade BSGs from 1980 onwards by MRI or histology were included. Those including mixed participant groups (for example adult and pediatric patients) were included if pediatric outcomes were reported separately. Studies had to report one of the following survival outcomes: one, two, three, five and/or greater than 5 year survival and median survival. To reduce the potential problems of publication bias introduced by very small studies, individual studies had to include a minimum of ten participants.

### Identification of trials

Database searches included MEDLINE, EMBASE, SCOPUS, The Cochrane Central Register of Controlled Trials (CENTRAL) and trial registers from the year 1980 onwards. The initial search was performed in February 2015 and updated in September 2015 and January 2017. Other searches included reference lists of identified and key review articles, abstracts from major conferences, hand searches of journals that comprised the most frequent venues for publications in this area and grey literature searches for unpublished data were also included. Searches were performed without language restrictions and attempts were made to obtain a translated copy if indicated.

### Study selection

Study selection and data extraction was conducted in two stages:

- Two reviewers independently assessed the title and abstract for the studies using the inclusion and exclusion criteria (H.H, A.P, A.F, S.R and S.H). Discrepancies of studies potentially included in the review were addressed and those unresolved were referred to an independent assessor (R.P).
- Data was extracted by a researcher using a standardized form (H.H) and 50% was independently checked by a second person (A.P, S.H). When further information was required, the author of the paper was contacted.

The study selection process and data extraction was piloted by applying the search strategy to a sample of 100 papers in order to check that the correct papers would be identified, interpreted and analyzed. The pilot study was used to refine the inclusion criteria to ensure that it could be applied consistently and that the correct data was extracted. The protocol published on the PROSPERO website was amended to include the modified inclusion criteria.

### Quality assessment

The Newcastle-Ottawa scale was adapted to assess the quality of studies as summarized in Table 1.

**Table 1:** Modified Newcastle-Ottawa Scale (adapted from [15])

Domain	Outcome assessed	
Selection	Representativeness of the exposed cohort (one point) Were BSG patients representative of the BSG patient that is typical in neurooncology practice?	Ascertainment of exposure (one point) Did the study specify where the information confirming diagnosis was taken from?
Comparability	Study controls for any additional factor (one point)	
Outcome	Was follow-up long enough for outcomes to occur (one point) Did follow up occur for at least 1 year?	Adequacy of follow up of cohorts (one point) Did the study account for all participants when assessing outcome of interest?

### Data synthesis

Meta-analysis of the subgroups high grade BSGs survival outcomes was performed using a random-effects model of logit-transformation proportions as proposed by Simmonds et al. [13].

We planned to assess if outcomes varied by specific subgroups (DIPG vs. high grade BSGs, age less than 3 years and greater than 3 years, duration of symptoms less than 6 months and above 6 months, K27 M H3.3 mutations and wild type H3.3 in DIPGs and AVCR1 mutations vs. non AVCR1 mutations). Where direct analyses had been performed in contributing studies, we planned to compare the groups by performing a meta-analysis of pooled hazard ratios using a random-effects model. Where alternative data was provided, we intended to transform results into estimates of hazard ratios using a previously describe schema [14].

For all results a p value of <0.008 were classed as statistically significant (according to Bonferroni correction for multiple outcomes) and calculated using the non-paired t-test. Heterogeneity was explored through consideration of study populations, study quality, predictor variables, and assessed in statistical terms using the I-squared (I-sq) statistic.

## Results

### Study selection

**Fig. 1:**
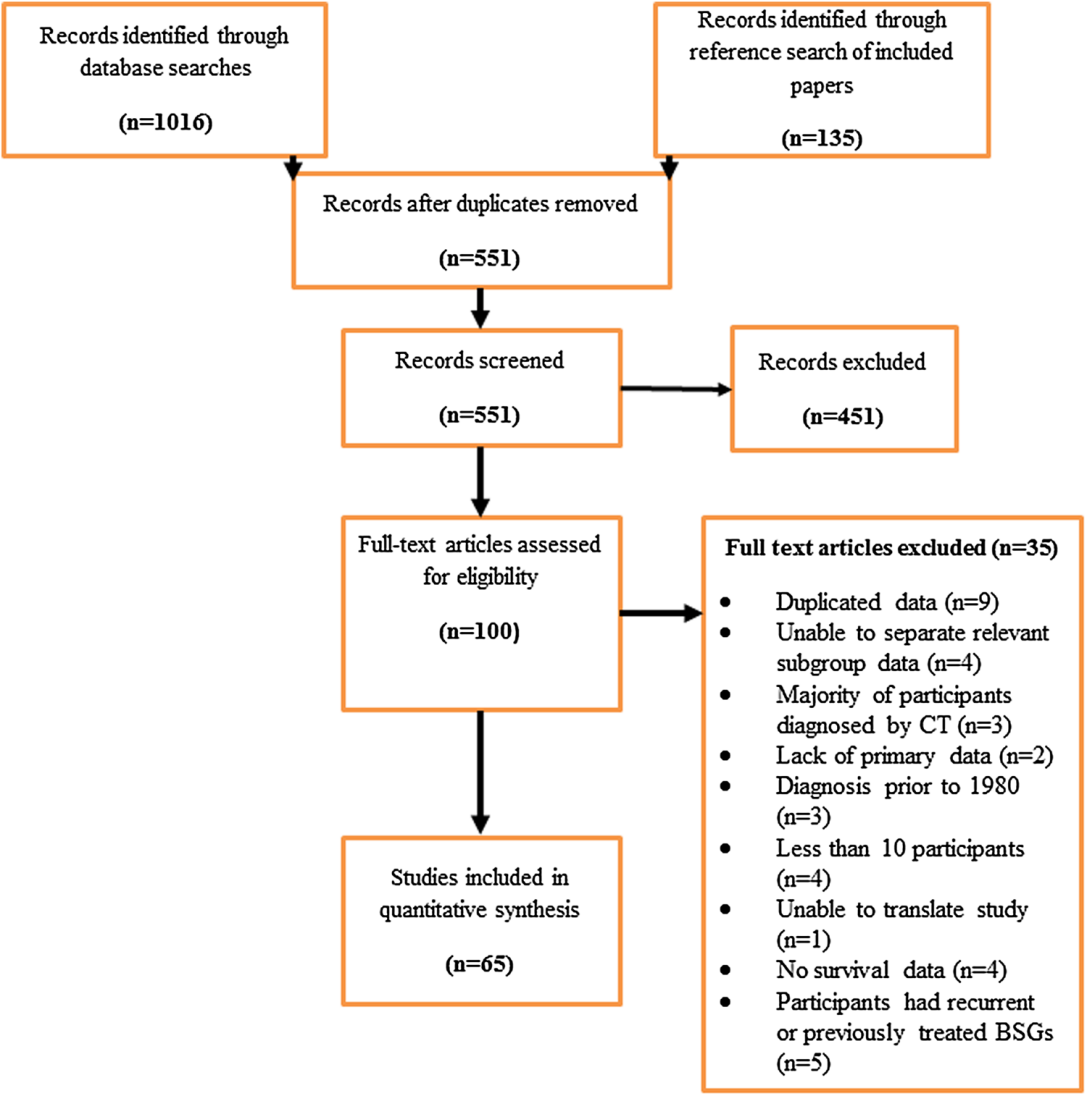
Summary of the screening process

We identified 1016 records through all electronic strategies mentioned and a further 143 potentials from reference searching. Six hundred duplicated records were removed leaving 551 papers for title, key word and abstract screening (Fig. 1). Following relevance screening a total of 100 papers were initially identified for full review. Forty four papers satisfied the inclusion criteria and a further 21 papers were identified by a comprehensive review of the references of included papers. This resulted in a total of 65 papers satisfying the eligibility criteria (see Fig. 1). The majority of studies were identified from the initial and updated searches (n = 64). Only two studies were identified through weekly electronic search updates [16, 17]. Included studies consisted of prospective cohorts (n = 42), retrospective cohorts (n = 23), case-controlled studies (n = 8) or randomized controlled trials (n = 3). Controls used in the case-controlled series included participants identified from a review article, matched cohorts, historical controls, participants included in a pilot study, and participants with non-brain stem diagnoses (for example ‘untreated regular and anaplastic astrocytoma’). The studies included in the systematic review represented 65 unique data sets (summary enclosed in supplementary file 1 See Supplementary Material). Thirty five papers were excluded following full text review for the following indications: duplicated data (n = 9), unable to separate data for relevant subgroup required (n = 4), majority of participants diagnosed via CT (n = 3), lack of primary data (n = 2), diagnosis prior to 1980 (n = 3), fewer than ten relevant participants (n = 4), participants had recurrent or previously treated brain stem gliomas (n = 5), no reported survival data (n = 4), unable to translate paper (written in Polish, n = 1).

### Quality of included studies

There is no currently accepted tool to evaluate the quality of prognostic studies, and the decision was made to use the Newcastle–Ottawa Scale [15]. This has three domains: selection, comparability and outcome. In this setting, “selection” refers to the representativeness of the patients within the study of the population to which it is drawn; “comparability” assesses any differences between groups beyond the identified potentially prognostic feature, and “outcome” refers to how complete and unbiased the assessment of outcomes is in the study. A summary of the quality of studies is included in the supplementary file 2 (See Supplementary Material).

### Selection quality

37.9% of included studies documented where the information was taken from. All studies (100%) were awarded a point for the representativeness of exposed cohort.

### Comparability quality

30.3% of included studies adjusted for additional factors. This included grade, prognostic factors, intervention and diagnostic measures.

### Outcome assessment quality

The majority of studies reported the duration and adequacy of follow up (92.44 and 71.2% respectively).

### Pooled estimates of survival

**Fig. 2:**
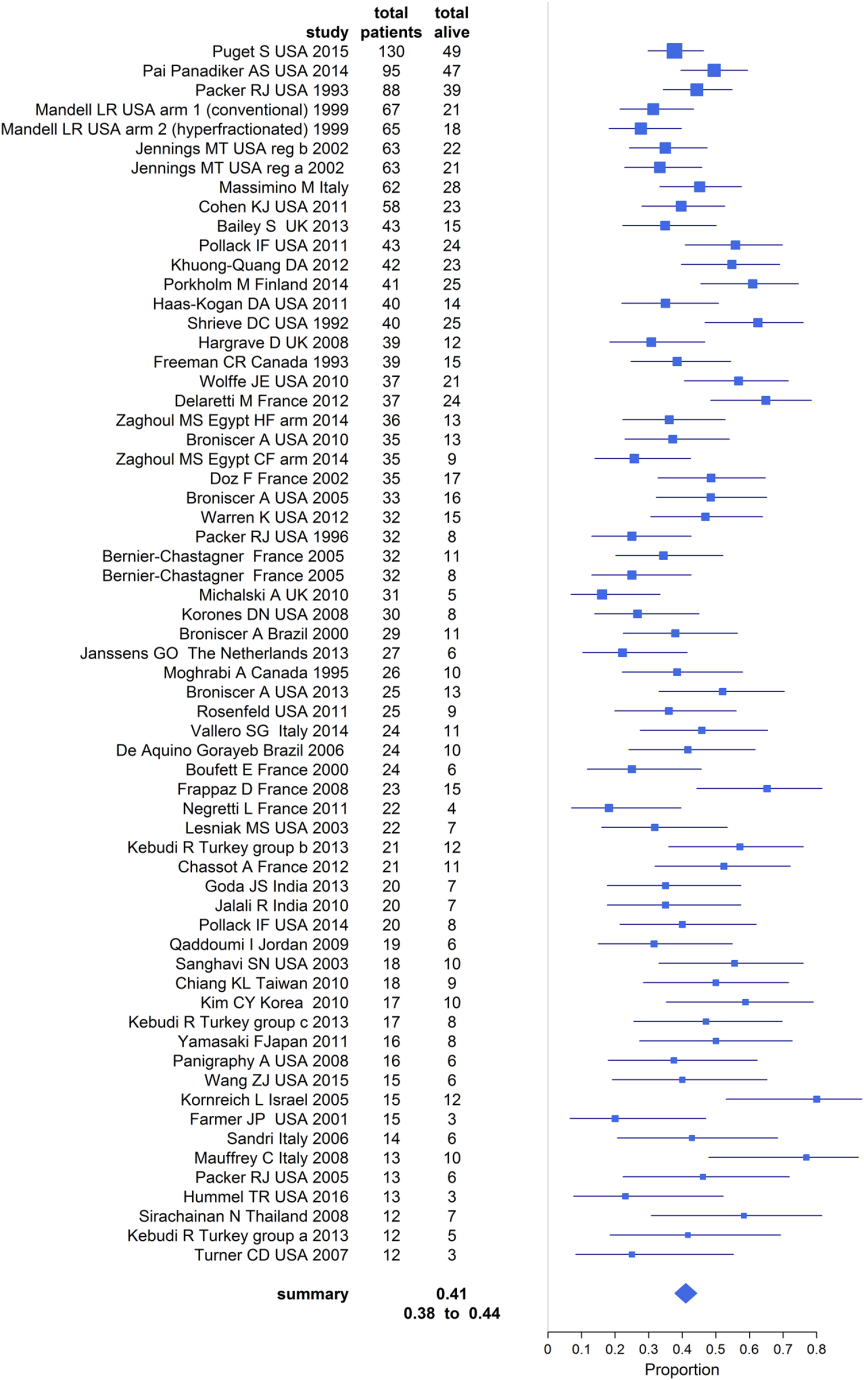
Pooled estimate of overall survival at 1 year

Overall survival could be estimated for 1, 2, and 3 year duration of follow-up. Appropriate 1 year overall survival was supplied in 63 data sets (2083 participants, Fig. 2) and estimated at 41% (95% confidence interval 38–44%, I-sq 52%).

Meta-analysis of 2 year overall survival was 15.3% (95% confidence interval 12–20%, I-sq 71.3%) based on 40 data sets (1329 participants).

Meta-analysis of 3 year overall survival demonstrated only 7.3% surviving (95% confidence interval 5.2–10%, I-sq 25.6%) from 15 data sets (584 participants).

Only four data sets reported 5 year overall survival, and we therefore did not perform a meta-analyses. There was significant heterogeneity of the variance measures reported with median overall survival results and the majority of studies reported the range of data analysed. Due to the lack of reported variance measures of median overall survival, we were unable to complete a meta-analysis of median overall survival results.

### Subgroup analyses

**Fig. 3:**
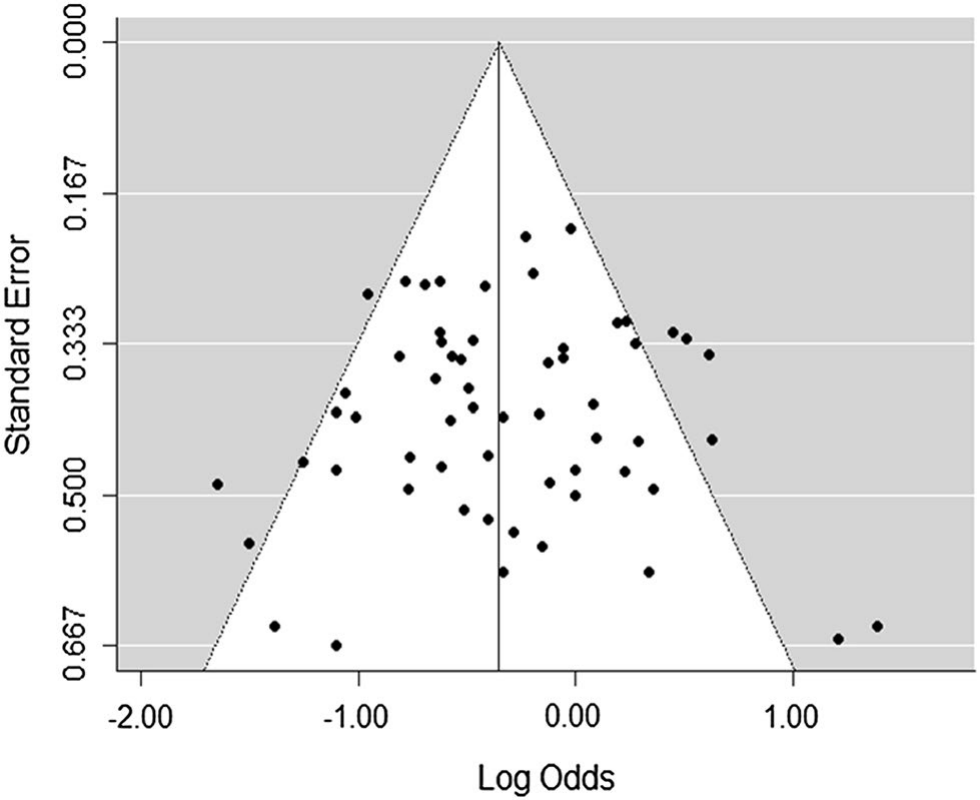
Funnel plot of total included studies reporting 1 year overall survival

Subgroup analysis is summarized in Table 2. We were unable to demonstrate any statistically significant results for temozolomide (Fig. 3), dose of radiotherapy, study-type, quality of study, mid-point date of study, classification of BSG as described in the studies i.e. DIPG versus other descriptions (including diffuse pontine glioma, diffuse brain stem gliomas, diffuse intrinsic brain stem gliomas, intrinsic pontine gliomas, intrinsic brain stem gliomas and high grade brain stem gliomas). Due to the lack of consistent data reporting in studies, we were unable to perform meta-analyses according to age of patients, duration of symptoms prior to presentation, specific clinical features or K27M and AVCR1 histone mutations.

**Table 2:** Overall survival at 1 year and subgroup analysis

Subgroup	Number of participants*	1 year OS (%) (95% CI, I-sq (%))	Difference (%), (95% CI, p = value)
Classification			
DIPGs	8018	40 (36–44% I-sq 48.5)	1.4 (−9.3 to 4.9%, p = 0.5)
Other high grade BSGs	1065	42 (37–47% I-sq 55.6)	
Drugs (DIPGs only)			
Temozolomide	202	42.7 (30–50%, 0)	1.2 (−6.2 to 17.1%, p = 0.3)
Non-Temozolomide	755	39 (34–44%, 54.1)	
Phase of study (DIPGs only)			
Phase 1 & 2 studies	295	37.6 (32–43%, 0)	4.5 (−13.3 to 3%, p = 0.2)
Non-phase 1 & 2 studies	723	42.1 (36–49%, 50.5)	
Radiotherapy (DIPGs only)			
Conventional radiotherapy only	235	41.3 (32–52% 52.5)	1.6 (−13 to 12.3%, p = 0.1)
Other interventions	783	39.7 (35–45% 47.6)	
Midpoint study entry (DIPGs only)			
Before 2006	390	37.9 (30–47% 59.9)	1 (−10.1 to 11.2%, p = 0.9)
From 2006	626	36.9 (31–43% 17.8)	

*Due to the lack of censoring information and IPD data all survival outcome percentages were calculated on the total number of participants included in the study rather than the number at each time point

### Publication bias

Publication bias for 1 year OS was assessed using the Sterne and Egger method and displayed in a funnel plot (Fig. 3). This was visually inspected, and there is no clear evidence of publication bias.

## Discussion

To our knowledge this is the first comprehensive systematic review of the literature that has attempted to summarize survival outcomes in those diagnosed with high grade BSGs. We performed a systematic search of 1151 citations, and included 66 papers (2279 participants) which confirmed very poor overall survival for these tumors. The pooled results should be interpreted cautiously: confidence intervals for the point estimates are wide and there is moderate heterogeneity. There was insufficient data to perform pooled analyses on 5 year and greater than 5 year OS.

Gliomas are graded according to the histological WHO classification of brain tumors [18], whereas BSGs are diagnosed according to an MRI classification since the publication of the article by Barkovich et al. [3] which was based on three clinical trials (87 participants). The use of different phrases across the studies to describe potentially similar brain stem gliomas was noted (for example ‘DIPG’, ‘diffuse pontine glioma’ and ‘intrinsic pontine glioma’). Although attempts to refer to the diagnostic criteria for inclusion in studies were reviewed to attempt to re-describe subgroup tumors consistently according to unified criteria [3] it was not always clear whether different phrases were describing the same subpopulation of gliomas. Our analysis looking at studies clearly describing DIPG versus those using other phrases to refer to diffuse/high-grade brain stem glioma suggest there is no clinically meaningful difference. However, the syntheses do demonstrate marked between-study heterogeneity. This may be due to an intrinsic heterogeneity in the tumor population within classification groups. For example, the histological grade of DIPGs varies from grade 2–4 and may have variable prognostic outcomes [19] although other authors did not demonstrate any clear difference in outcomes in analyses of histological grade [11, 20]. High grade BSGs may also include low grade features which have more favorable outcomes, for example exophytic extensions which is deemed a low grade feature but may be present in gliomas classified as high grade. The varying definitions of diffuse pontine gliomas, alongside the other classifications of BSGs and true biological variation in grading may contribute to the heterogeneity in meta-analysis, although between-study heterogeneity was not reduced when the alternative descriptions used by the authors were evaluated.

Subgroup meta-analyses of studies reported as DIPGs revealed similar 1–2 year OS outcomes, with no clear differences seen over time, with the use of chemotherapy or different radiotherapy regimes. The lack of an observed difference should not be interpreted as evidence of a lack of effect of these interventions: the estimates are indirect, have wide confidence intervals and moderate unexplained heterogeneity.

Due to the lack of comparable prognostic data we were unable to perform meta-analyses of outcomes according to age, duration of symptoms, K27M histone and ACVR1 mutations. We were also unable to perform pooled analyses of median overall survival due to the lack of variance measures reported.

Studies included in the analysis included prospective, retrospective, case-controlled cohort and non-blinded randomized-controlled trials. The different study designs may contribute to the heterogeneity, unfortunately, we were unable to undertake a subgroup analysis due to the small number of particular study designs (only three randomized-controlled studies were included).

We attempted to understand the source of the between-study heterogeneity by subgroup analysis according to the overall score allocated using the Newcastle–Ottawa quality assessment tool; this did not indicate the quality of the study may have contributed to the heterogeneity. Attrition and reporting biases (including publication biases, selective outcome and selective analysis reporting) were also considered to see if they could contribute to heterogeneity reported. Visual inspection of the funnel plots of 1 year OS results using the Sterne and Egger method [21] (Fig. 3) did not show any clear evidence of publication bias. Missing data is a significant limiting factor in this review, both reducing precision, the possibility of specific subgroup analyses, and remaining uncertainty about heterogeneity because of lack of clear information about the flow of patients through each stage of the studies.

## Applicability

Our review identified serious inconsistencies in how the classifications of BSGs are reported. We found that they are defined using MRI, WHO grading or both making it difficult to group results with confidence. A validated uniform diagnostic tool is required to limit the heterogeneity of results analysed. As MRI classification appears to include gliomas of differing grades, performing biopsies on all diagnoses would improve accuracy and possibly, give better indications of outcomes [22].

Systematic reviews have been hampered with inconsistent outcome measures reporting in the past which resulted in the Core Outcome Measure in Effectiveness Trials (COMET) initiative. This aims to develop standardised outcomes known as ‘core outcome sets’ to reduce heterogeneity and difficulties in undertaking reviews. Inconsistent definitions have also been previously reported in paediatric supportive care [23] which led to a study using the Delphi method to develop variables and outcomes to be used globally. While the COMET initiative is primarily concerned with interventional studies, a similar process to create a uniform core set of definitions and outcome variables for paediatric brain stem gliomas would enable better analysis of studies and would reduce heterogeneity. Greater transparency within studies is required with clear documentation of study methodology and protocol publication is required to minimise bias.

This systematic review is limited by the lack of adequate reporting of censored and missing participants. This could be overcome with mechanisms for sharing individual participant data (IPD) in future studies. The value of IPD in interventional studies has been demonstrated in numerous meta-analyses and its use has increased over the past decade.

The use of IPD in future systematic reviews would enable more consistent inclusion and exclusion criteria, account for missing data, verify published and use unpublished results, would allow development of prognostic models and would reduce study heterogeneity. However, IPD analyses are labour intensive and time consuming. There are also concerns regarding the ethical implications of patient confidentiality, although this can be overcome by ensuring no patient identifiable data is accessible. A further problem that may be encountered is that study authors may not be contactable or willing to contribute which may result in biased meta-analyses. It is also important to remember that the quality of meta-analyses from IPD is dependent on the quality of studies included.

If studies do not supply IPD then clear information regarding censoring (how many and exact time), number at risk, clear documentation of measures of variance and when outcomes are assessed from should be supplied.

## Conclusion

Survival from high-grade brain stem gliomas in childhood remains very poor, with this systematic review estimating that only four in ten young people diagnosed with a DIPG will be alive at one year after diagnosis. The studies assessed do not clearly demonstrate an improvement over time, or show any major impact of chemotherapy or alternative radiotherapy approaches. There were marked differences between the studies which were not clearly explained as chance variation, differences in the quality of the study report, the type of study (e.g. phase I/II), or the exact classification of the tumors included. Commonly proposed prognostic features, such as age, duration of symptoms, and newer biological predictors, K27M histone and ACVR1 mutations, could not be assessed through insufficient reporting of this information.

Better understanding of how to predict outcomes from this rare group of pediatric brain tumors will require harmonized and collaborative collection of data, pooled at an individual patient level, driven by a desire to develop new predictors and assess the validity of previously proposed factors. As a coordinated global community, those involved in pediatric neuro-oncology are ideally placed meet this challenge.

## Electronic supplementary material

Below is the link to the electronic supplementary material.

Supplementary material 1 (DOCX 23 KB)

Supplementary material 2 (DOCX 139 KB)

## Abbreviations

DIPG: Diffuse intrinsic pontine glioma
BSG: Brain stem glioma
RCT: Randomised-controlled trial
CNS: Central nervous system
WHO: World Health Organisation
MRI: Magnetic resonance imaging
CENTRAL: The Cochrane Central Register of Controlled Trials
OS: Overall survival
USA: United States of America
UK: United Kingdom
