# Supplementary material for: Survival rates and prognostic predictors of high grade brain stem gliomas in childhood: a systematic review and meta-analysis
Source: J Neurooncol. 2017 Jul 5;135(1):13–20. doi: 10.1007/s11060-017-2546-1 (PMC5658459; doi:10.1007/s11060-017-2546-1)
Supplement: Supplementary file 1 — Supplementary material 1 (DOCX 23 KB) [file 11060_2017_2546_MOESM1_ESM.docx]

Supplementary file 2

**Table 4: Summary of Newcastle-Ottawa Scale points allocation for cohort studies**

Green: maximum number of points awarded

Yellow: 1 out of 2 points awarded

Red: no points awarded

| **Author/year/location** | **Study type** | **Representation of exposed cohort** | **Representativeness of non-exposed cohort** | **Comparability of cohorts of additional factors** | **Was follow-up long enough?** | **Adequacy of follow-up** |
| --- | --- | --- | --- | --- | --- | --- |
| Warren K 2012 USA | Case-controlled |  |  |  | * | * |
| Pollack IF 2011 USA | Cohort | * | N/A |  | * | * |
| Kim CY 2010 Korea | Cohort | * | N/A |  |  |  |
| Pollack IF 2014 USA | Cohort | * | N/A |  | * | * |
| Farmer JP 2001 USA | Cohort | * | N/A |  | * | * |
| Goda JS 2013 India | Cohort | * | N/A |  | * | * |
| Sirachainan N 2008 Thailand | Cohort | * | N/A |  | * | * |
| Hargrave D 2008 UK | Cohort | * | N/A | * | * | * |
| Delaretti M 2011 France | Cohort | * | N/A |  | * |  |
| Sandri 2006 Italy | Cohort | * | N/A |  | * | * |
| Delaretti M 2012 France | Cohort | * | N/A | * | * |  |
| Vallero SG 2014 Italy | Cohort | * | N/A |  | * | * |
| Bailey S 2013 UK | Cohort | * | N/A |  | * | * |
| Qaddoumi I 2009 Jordan | Cohort | * | N/A |  | * | * |
| Massimino M 2008 Italy | Cohort | * | N/A |  | * |  |
| Lober RM 2014 USA | Cohort | * | N/A | * | * | * |
| Pai Panadiker AS 2014 USA | Cohort | * | N/A | * | * | * |
| Freeman CR 1993 Canada | Cohort | * | N/A | * | * | * |
| Packer RJ 1993 USA | Cohort | * | N/A | * | * | * |
| Shrieve DC 1992 USA | Cohort | * | N/A | * | * |  |
| Negretti L 2011 France | Cohort | * | N/A |  | * |  |
| Janssens GO 2013 The Netherlands | Matched-cohort | * | * | * | * |  |
| Wolff JE 2010 USA | Case-controlled | * |  | * | * | * |
| Yamasaki F 2011 Japan | Cohort | * | N/A | * | * | * |
| Steffen-Smith 2014 USA | Cohort | * | N/A | * | * |  |
| Puget S 2015 France | Cohort | * | N/A | * |  |  |
| Bradley KA 2013 USA | Case- controlled | * |  |  | * |  |
| Mauffrey C 2006 Italy | Cohort | * | N/A | * | * |  |
| Kebudi R 2013 Turkey | Cohort | * | N/A |  | * |  |
| Broniscer A 2013 USA | Cohort | * | N/A | * | * |  |
| Broniscer A 2010 USA | Cohort | * | N/A | * | * |  |
| Haas-Kogan DA 2011 USA | Cohort | * | N/A |  | * | * |
| Jalali R 2010 India | Cohort | * | N/A | * | * | * |
| Hipp SJ 2011 USA | Cohort | * | N/A | * | * | * |
| Porkholm M 2014 Finland | Case-controlled | * | N/A |  | * | * |
| Broniscer A 2000 Brazil | Cohort | * | N/A |  | * | * |
| Chassot A 2012 France | Cohort | * | N/A |  | * | * |
| Broniscer A 2005 USA | Cohort | * | N/A |  | * | * |
| Chiang KL 2010 Taiwan | Double-arm cohort | * | N/A |  | * |  |
| Lesniak MS 2003 USA | Cohort | * | N/A |  | * | * |
| Cohen KJ 2011 USA | Case-controlled | * |  |  | * | * |
| Michalski A 2010 UK | Case-controlled | * |  | * | * | * |
| Bernier-Chastagner 2005 France | Cohort | * | N/A |  | * | * |
| de Aquino Gorayeb 2006 Brazil | Cohort | * | N/A | * | * | * |
| Korones DN 2008 USA | Cohort | * | N/A |  | * | * |
| Packer RJ 1996 USA | Cohort | * | N/A |  | * | * |
| Rosenfeld A 2011 USA | Cohort | * | N/A |  | * | * |
| Doz F 2002 France | Cohort | * | N/A |  | * | * |
| Kornreich L 2005 Israel | Cohort | * | N/A | * | * | * |
| Panigraphy A 2008 USA | Case-controlled | * |  |  | * | * |
| Allen J 1999 USA | Cohort | * | N/A |  | * | * |
| Khuong-Quang DA 2012 | Cohort | * | N/A | * |  |  |
| Frappaz D 2008 France | Case-controlled | * |  | * | * | * |
| Packer RJ 2005 USA | Cohort | * | N/A |  | * | * |
| Moghrabi A 1995 Canada | Cohort | * | N/A |  | * | * |
| Sanghavi SN 2003 USA | Cohort | * | N/A |  | * | * |
| Marcus KJ 2003 USA | Cohort | * | N/A |  |  |  |
| Bradley KA 2008 USA | Cohort | * | N/A |  |  |  |
| Pirotte BJ 2007 Belgium | Cohort | * | N/A |  | * | * |
| Turner CD 2007 USA | Cohort | * | N/A |  | * | * |
| Wang ZJ 2015 USA | Cohort | * | N/A |  | * | * |
| Boufett France 2000 | Cohort | * | N/A |  | * | * |
| Mandell LR 1999 USA | Non-blinded RCT | * | N/A |  | * | * |
| Jennings MT 2002 USA | Non-blinded RCT | * | N/A | * | * |  |
| Zaghoul MS 2014 Egypt | Non-blinded RCT | * | N/A |  | * |  |
| Hummel TR 2016 USA | Cohort | * | N/A |  | * | * |
