# Supplementary material for: Survival rates and prognostic predictors of high grade brain stem gliomas in childhood: a systematic review and meta-analysis
Source: J Neurooncol. 2017 Jul 5;135(1):13–20. doi: 10.1007/s11060-017-2546-1 (PMC5658459; doi:10.1007/s11060-017-2546-1)
Supplement: Supplementary file 2 — Supplementary material 2 (DOCX 139 KB) [file 11060_2017_2546_MOESM2_ESM.docx]

| **Author/year/**  **location** | **Phase 1/2** | **Prospective/**  **retrospective** | **Study type** | **Classification** | **Number of participants** | **Age group** | **Follow up** |
| --- | --- | --- | --- | --- | --- | --- | --- |
| Warren K 2012 USA[1] | 2 | Prospective | Case-controlled | DIPG | 32 | 1.8-14.8 years | All participants accounted for.  Duration of follow up unclear. |
| Pollack IF 2011 USA [2] | 2 | Prospective | Cohort | Diffuse intrinsic BSG | 43 | 3.4-18.7 years | All participants accounted for.  Participants followed for at least 1 year in the absence of disease progression or dose limiting toxicity. |
| Kim CY 2010 Korea [3] |  | Prospective | Cohort | DIPG | 17 | 3-16 years | All participants accounted for.  Patients followed up for at least 1 year or until death. |
| Pollack IF 2014 USA [4] |  | Prospective | Cohort | DIPG  HGG | 20 | 1 and 21 years | All participants accounted for.  Follow up not specified, although evaluations obtained at 12 week intervals |
| Farmer JP 2001 USA [5] |  | Retrospective | Cohort | Diffuse pontine glioma | 15 | 1-11 years. | All participants accounted for.  Patients followed up until death. |
| Goda JS 2013 India [6] | 2 | Prospective | Cohort | DIPG | 20 | Age range not specified | All participants accounted for.  Patients followed up until death. |
| Sirachainan N 2008 Thailand [7] |  | Prospective | Cohort | DIPG | 12 | 2.8-9.1 years | All participants accounted for.  Median follow-up time 14.5 months |
| Hargrave D 2008 UK [8] |  | Retrospective | Cohort | DIPG | 39 | 0.2-14.7 years | All participants accounted for.  Participants followed up until death or minimum of 15 months. |
| Delaretti M 2011 France [9] |  | Retrospective | Cohort | Diffuse intrinsic BSG | 44 | Mean age 6 years.  No other information supplied. | Sixteen participants alive at last follow-up.  Follow-up ranged from 4 days to 246 months after biopsy (mean 46 months). |
| Sandri A 2006 Italy [10] |  | Retrospective | Cohort | Diffuse pontine glioma | 14 | 2.4-11.4 years | All participants accounted for.  Follow up occurred until death or minimum of 8 months. |
| Delaretti M 2012 France [11] |  | Retrospective | Cohort | Diffuse BSG | 37 | 2-12 years | All participants not accounted for.  Duration of follow-up unknown. |
| Vallero SG 2014 Italy [12] |  | Retrospective | Cohort | DIPG | 24 | 2.9-16.6 years | All participants accounted for.  Follow up occurred until death or minimum of 9 months. |
| Bailey S 2013 UK [13] | 2 | Prospective | Cohort | DIPG | 43 | 2-20 years | All participants accounted for.  Follow up until occurred death or minimum of 14 months. |
| Qaddoumi I 2009 Jordan [14] |  | Prospective | Cohort | Diffuse pontine glioma | 19 | 2.1-16.2 years | All participants accounted for.  Two participants lost to follow up at 2.2 and 7 months. All other patients followed up until death or minimum of 16 months. |
| Massimino M 2008 Italy [15] |  | Prospective | Cohort | Diffuse pontine glioma | 62 | 3-15 years | All participants accounted for.  Follow up until death or minimum of 31 months. |
| Lober RM 2014 USA [16] |  | Retrospective | Cohort | DIPG | 21 | 2.3-13.2 years | All participants accounted for.  All participants followed up until death apart from 1 participant alive at end of study. |
| Pai Panadiker AS 2014 USA [17] |  | Retrospective | Cohort | DIPG | 95 | 1.5-16.5 years | All participants accounted for.  All patients followed up until death or a minimum of 47 months. |
| Freeman CR 1993 Canada [18] | 1/2 | Prospective | Cohort | DPG | 39 | 3-15 years | All participants accounted for.  All participants followed up until death or minimum of 12 months. |
| Packer RJ 1993 USA [19] | 1/2 | Prospective | Cohort | Diffuse and  malignant BSGs | 88 | 2-20 years | All participants accounted for.  All participants followed up until death apart from 2 participants alive at end of study (but unknown duration of follow-up). |
| Shrieve DC 1992 USA [20] |  | Retrospective | Cohort | Diffuse and  high grade BSGs | 40 | Less than the age of 18 years.  No other information supplied. | All participants accounted for.  All participants followed up until death apart from 2 participants alive at end of study. |
| Negretti L 2011 France [21] |  | Prospective | Cohort | DIPG | 22 | 2.9-12.5 years | 1 participant not accounted for.  All patients followed up until death apart from 1 participant (unknown duration of follow up). |
| Janssens GO 2013 The Netherlands [22] |  | Prospective | Matched-cohort | DIPG | Hypofractionated radiotherapy: 27.  Standard radiotherapy: 27 | 3-14 years | All participants accounted for.  All participants followed up until death apart from 1 participant who was followed up for 2.4 years. |
| Wolff JE 2010 USA [23] |  | Prospective | Case-controlled | DIPG | 37 | 3.22-17.93 years | All participants accounted for.  All participants followed up until death apart from 1 participant who was followed up for 4.5 years. |
| Yamasaki F 2011 Japan [24] |  | Retrospective | Cohort | DIPG | 16 | 3-24 years | All participants not accounted for.  All participants followed up until death or last follow-up. |
| Steffen-Smith 2014 USA [25] |  | Retrospective | Cohort | DIPG | 75 | 2-17 years | All participants not accounted.  All participants followed up until death or last follow-up (unknown). |
| Puget S 2015 France [26] |  | Prospective | Cohort | DIPG | 130 | 2.4 to 16.4 years | All participants not accounted for.  All participants followed up until death, unknown number of participants not accounted (either alive at completion of study or lost to follow-up). |
| Bradley KA 2013 USA [27] | 2 | Prospective | Case-controlled | Intrinsic pontine glioma | 60 | 1.7-17.3 years | All participants not accounted for.  All participants followed up for a minimum of 1 year but unknown number of participants not accounted (either alive at completion of study or lost to follow-up).  . |
| Mauffrey C 2006 Italy [28] |  | Retrospective | Cohort | Diffuse pontine glioma | 13 | Mean age of presentation in group 1: 7.1 years.  Mean presentation in group 2: 6.7 years.  Age range not supplied. | All participants accounted for.  All participants followed up until death or last follow-up (unknown). |
| Kebudi R 2013 Turkey [29] |  | Retrospective | Cohort | DIPG | 50 | 6 months-16 years | All participants accounted for.  All participants followed up until death or minimum of 3 years. |
| Broniscer A 2010 USA [30] | 1 | Prospective | Cohort | DIPG | 35 | 2.8-16.4 years | All participants accounted for.  All participants followed up until death or last follow-up (unknown). |
| Broniscer A 2013 USA [31] | 1 | Prospective | Cohort | DIPG | 25 | 2.3-17.2 years | All participants accounted for.  All participants followed up until death or minimum of 15 months. |
| Haas-Kogan DA 2011 USA [32] | 2 | Prospective | Cohort | DIPG | 40 | 3.3-16.5 years | All participants accounted for.  All participants followed up until death or minimum of 12 months. |
| Hipp SJ 2011 USA [33] |  | Prospective | Cohort | DIPG | 34 | 1.6-14.6 years | All participants not accounted for.  Unknown number of participants followed up until death or last follow up. |
| Jalali R 2010 India [34] |  | Prospective | Cohort | DIPG | 20 | 3-18 years | All participants accounted for.  All participants followed up until death or minimum of 3 years. |
| Porkholm M 2014 Finland [35] |  | Prospective | Case-controlled | DIPG | 41 | 1-16 years | All participants accounted for.  33 participants followed up until death  8 alive at end of study (unknown duration of follow up but followed for a minimum of 5 months) |
| Broniscer A 2000 Brazil [36] |  | Prospective | Cohort | Diffuse BSG | 29 | 1.1-13.8 years | All participants accounted for.  21 patients followed up until death.  8 alive at end of study (but unknown duration of follow up). |
| Chassot A 2012 France [37] |  | Prospective | Cohort | DIPG | 21 | 4-16 years | 20 participants accounted for.  16 participants followed until death or for a minimum of 11 months.  1 patient unaccounted |
| Broniscer A 2005 USA [38] |  | Prospective | Cohort | Diffuse BSG | 33 | 3.1-15.2 years | All participants accounted for.  All participants followed up until death. |
| Chiang KL 2010 Taiwan [39] |  | Retrospective | Double-arm cohort | Diffuse BSG | 18 | 4.5-13.4 years | 3 participants excluded at start of study as lost to follow up.  All other participants accounted for  Participants followed up until death or followed up for a minimum of 5 months |
| Lesniak MS 2003 USA [40] |  | Retrospective | Cohort | HGG | 12 | 3 months-20 years | All participants accounted for.  All participants followed up until death. |
| Cohen KJ 2011 USA [41] | 2 | Prospective | Case-control | DIPG | 58 | 3.3-16.2 years | All participants accounted for.  All participants followed up until death apart from 1 patient who was lost to follow up at 25 months. |
| Michalski A 2010 UK [42] |  | Prospective | Case-controlled | DIPG | 31 | 1.83-16.2 years | All participants accounted for.  All participants followed up until death or a minimum of 1 year. |
| Mandell LR 1999 USA [43] |  | Prospective | Non-blinded RCT | Diffuse intrinsic BSG | Conventional radiotherapy: 67.  Hyperfractionated radiotherapy: 65. | 3.3-17.6 years | 5 participants lost to follow-up at 6, 13, 17, 31 and 44 months.  All other participants followed up until death or last follow up (unknown number of participants) and duration. |
| Bernier-Chastagner 2005 France [44] | 2 | Prospective | Cohort | Diffuse BSG | 32 | 3-16.8 years | All participants accounted for.  All participants followed up until death. |
| de Aquino Gorayeb 2006 Brazil [45] |  | Retrospective | Cohort | Diffuse BSG | 24 | 1-14 years | All participants accounted for.  All participants followed up until death or a minimum of 4 months. |
| Korones DN 2008 USA [46] | 2 | Prospective | Cohort | DIPG | 30 | 3-14 years | All participants accounted for.  All participants followed up until death |
| Packer RJ 1996 USA[47] | 1/2 | Prospective | Cohort | Diffuse intrinsic BSG | 32 | 2-17 years | All participants accounted for.  All participants followed up until death. |
| Rosenfeld A 2011 USA [48] |  | Retrospective | Cohort | Diffuse intrinsic BSG | 25 | 2-16 years | All participants accounted for.  All participants followed up until death. |
| Doz F 2002 France [49] |  | Prospective | Cohort | Not specified | 35 | 3-15 years | All participants accounted for.  All participants followed up until death. |
| Kornreich L 2005 Israel [46] |  | Retrospective | Cohort | Diffuse pontine glioma | 15 | 2-19 years | All participants accounted for.  All participants followed up until death or a minimum of 2 years. |
| Panigraphy A 2008 USA [50] |  | Retrospective | Case-controlled | Diffuse intrinsic BSG | 16 | 3.5-14.5 years | 1 participants excluded from analysis as lost to follow-up.  All other patients followed up until death. |
| Allen J 1999 USA [51] | 1/2 | Prospective | Cohort | DIPG and HGG | 34 | 3.6-15.4 years | All participants accounted for.  All followed up until death or a minimum of 3 years. |
| Khuong-Quang DA Canada 2012 [52] |  | Retrospective | Cohort | DIPG | 40 | 0-16.3 years | 3 participants unaccounted.  All other participants followed up until death or a minimum of 6 years |
| Frappaz D 2008 France [53] |  | Prospective | Case control | Diffuse intrinsic BSG | 23 |  | All participants accounted for.  All participants followed up until death. |
| Packer RJ 2005 USA [54] | 1 | Prospective | Cohort | Diffuse intrinsic BSG and malignant glioma | 13 | 3-12 years | All participants accounted for.  2 participants alive at end of the study. |
| Moghrabi A 1995 Canada [55] |  | Retrospective | Cohort | Diffuse BSG | 26 | 1.8-16.2 years | All participants accounted for.  All participants followed up until death or a minimum of 1 year. |
| Sanghavi SN 2003 USA [56] | 1 | Prospective | Cohort | DIPG and Intrinsic BSG | 13 | 0-14 years | Participants not accounted.  Unknown number of participants followed up until death or date of last follow up. |
| Marcus KJ 2003 USA [57] | 1 | Prospective | Cohort | DPG | 18 | 4-26 years | Participants not accounted for.  Unknown number of participants followed up until death or date of last follow up. |
| Bradley KA 2008 USA [58] | 1 | Prospective | Cohort | DIPG | 44 | 2-20 years | Participants not accounted for.  Unknown number of participants followed up until death or date of last follow up. |
| Jennings MT 2002 USA [59] | 2 | Prospective | Non-blinded RCT | Diffuse pontine glioma  Malignant glioma | Regimen A: 32  Regimen B: 31 | 3-15 years | All participants accounted for.  1 participant lost to follow-up. Other participants followed up for a minimum of 2 years. |
| Pirotte BJ 2007 Belgium [60] |  | Retrospective | Cohort | Intrinsic pontine glioma | 14 | 1-13 years | All participants accounted for.  All participants followed up until death or minimum duration of 1 year. |
| Turner CD 2007 USA [61] | 2 | Prospective | Cohort | DIPG | 12 | 2-9 years | All participants accounted for.  All participants followed up until death. |
| Wang ZJ 2015 USA [62] |  | Retrospective | Cohort | DIPG | 15 | 1-16 years | All participants accounted (1 lost to follow-up)  All other participants followed up until death. |
| Zaghloul MS 2014 Egypt [63] |  | Prospective | Non–blinded RCT | DIPG | Hypofractionated radiotherapy: 35  Conventional radiotherapy: 36 | Median age 7.9 years  Standard deviation for both groups supplied | All participants accounted.  All participants followed up until death or minimum of 8 months. |
| Hummel TR USA [64] |  | Prospective | Cohort | DIPG | 13 | 3-16 years | All participants followed up until death. |
| Bouffet E 2000 France [65] |  | Prospective | Cohort | DIPG | 35 | 3-18 years | All participants followed up until death. |

**References of included studies**

1. Warren K, Bent R, Wolters PL, Prager A, Hanson R, Packer R, Shih J, Camphausen K: **A phase 2 study of pegylated interferon alpha-2b (PEG-Intron((R))) in children with diffuse intrinsic pontine glioma**. *Cancer* 2012, **118**(14):3607-3613.

2. Pollack IF: **Multidisciplinary management of childhood brain tumors: A review of outcomes, recent advances, and challenges - A review**. *J Neursurg Pediatr* 2011, **8**(2):135-148.

3. Kim CY, Kim SK, Phi JH, Lee MM, Kim IA, Kim IH, Wang KC, Jung HL, Lee MJ, Cho BK: **A prospective study of temozolomide plus thalidomide during and after radiation therapy for pediatric diffuse pontine gliomas: preliminary results of the Korean Society for Pediatric Neuro-Oncology study**. *J Neurooncol* 2010, **100**(2):193-198.

4. Pollack IF, Jakacki RI, Butterfield LH, Hamilton RL, Panigrahy A, Potter DM, Connelly AK, Dibridge SA, Whiteside TL, Okada H: **Antigen-specific immune responses and clinical outcome after vaccination with glioma-associated antigen peptides and polyinosinic-polycytidylic acid stabilized by lysine and carboxymethylcellulose in children with newly diagnosed malignant brainstem and nonbrainstem gliomas**. *Journal of clinical oncology : official journal of the American Society of Clinical Oncology* 2014, **32**(19):2050-2058.

5. Farmer JP, Montes JL, Freeman CR, Meagher-Villemure K, Bond MC, O'Gorman AM: **Brainstem Gliomas. A 10-year institutional review**. *Pediatr Neurosurg* 2001, **34**(4):206-214.

6. Goda JS, Dutta D, Raut N, Juvekar SL, Purandare N, Rangarajan V, Arora B, Gupta T, Kurkure P, Jalali R: **Can multiparametric MRI and FDG-PET predict outcome in diffuse brainstem glioma? A report from a prospective phase-II study**. *Pediatr Neurosurg* 2013, **49**(5):274-281.

7. Sirachainan N, Pakakasama S, Visudithbhan A, Chiamchanya S, Tuntiyatorn L, Dhanachai M, Laothamatas J, Hongeng S: **Concurrent radiotherapy with temozolomide followed by adjuvant temozolomide and cis-retinoic acid in children with diffuse intrinsic pontine glioma**. *Neuro Oncol* 2008, **10**(4):577-582.

8. Hargrave D, Chuang N, Bouffet E: **Conventional MRI cannot predict survival in childhood diffuse intrinsic pontine glioma**. *J Neurooncol* 2008, **86**(3):313-319.

9. Dellaretti M, Touzet G, Reyns N, Dubois F, Gusmao S, Pereira JL, Blond S: **Correlation among magnetic resonance imaging findings, prognostic factors for survival, and histological diagnosis of intrinsic brainstem lesions in children**. *J Neurosurg Pediatr* 2011, **8**(6):539-543.

10. Sandri A, Sardi N, Genitori L, Giordano F, Peretta P, Basso ME, Bertin D, Mastrodicasa L, Todisco L, Mussa F *et al*: **Diffuse and focal brain stem tumors in childhood: prognostic factors and surgical outcome. Experience in a single institution**. *Childs Nerv Syst* 2006, **22**(9):1127-1135.

11. Dellaretti M, Reyns N, Touzet G, Dubois F, Gusmao S, Pereira JL, Blond S: **Diffuse brainstem glioma: prognostic factors**. *J Neurosurg* 2012, **117**(5):810-814.

12. Vallero SG, Bertin D, Basso ME, Pittana LS, Mussano A, Fagioli F: **Diffuse intrinsic pontine glioma in children and adolescents: a single-center experience**. *Childs Nerv Syst* 2014, **30**(6):1061-1066.

13. Bailey S, Howman A, Wheatley K, Wherton D, Boota N, Pizer B, Fisher D, Kearns P, Picton S, Saran F *et al*: **Diffuse intrinsic pontine glioma treated with prolonged temozolomide and radiotherapy--results of a United Kingdom phase II trial (CNS 2007 04)**. *European journal of cancer (Oxford, England : 1990)* 2013, **49**(18):3856-3862.

14. Qaddoumi I, Ezam N, Swaidan M, Jaradat I, Mansour A, Abuirmeileh N, Bouffet E, Al-Hussaini M: **Diffuse pontine glioma in Jordan and impact of up-front prognosis disclosure with parents and families**. *J Child Neurol* 2009, **24**(4):460-465.

15. Massimino M, Spreafico F, Biassoni V, Simonetti F, Riva D, Trecate G, Giombini S, Poggi G, Pecori E, Pignoli E *et al*: **Diffuse pontine gliomas in children: changing strategies, changing results? A mono-institutional 20-year experience**. *J Neurooncol* 2008, **87**(3):355-361.

16. Lober RM, Cho YJ, Tang Y, Barnes PD, Edwards MS, Vogel H, Fisher PG, Monje M, Yeom KW: **Diffusion-weighted MRI derived apparent diffusion coefficient identifies prognostically distinct subgroups of pediatric diffuse intrinsic pontine glioma**. *J Neurooncol* 2014, **117**(1):175-182.

17. Pai Panandiker AS, Wong JK, Nedelka MA, Wu S, Gajjar A, Broniscer A: **Effect of time from diagnosis to start of radiotherapy on children with diffuse intrinsic pontine glioma**. *Pediatr Blood Cancer* 2014, **61**(7):1180-1183.

18. Freeman CR, Krischer JP, Sanford RA, Cohen ME, Burger PC, del Carpio R, Halperin EC, Munoz L, Friedman HS, Kun LE: **Final results of a study of escalating doses of hyperfractionated radiotherapy in brain stem tumors in children: a Pediatric Oncology Group study**. *Int J Radiat Oncol Biol Phys* 1993, **27**(2):197-206.

19. Packer RJ, Boyett JM, Zimmerman RA, Rorke LB, Kaplan AM, Albright AL, Selch MT, Finlay JL, Hammond GD, Wara WM: **Hyperfractionated radiation therapy (72 Gy) for children with brain stem gliomas. A Childrens Cancer Group Phase I/II Trial**. *Cancer* 1993, **72**(4):1414-1421.

20. Shrieve DC, Wara WM, Edwards MS, Sneed PK, Prados MD, Cogen PH, Larson DA, Levin VA: **Hyperfractionated radiation therapy for gliomas of the brainstem in children and in adults**. *Int J Radiat Oncol Biol Phys* 1992, **24**(4):599-610.

21. Negretti L, Bouchireb K, Levy-Piedbois C, Habrand JL, Dhermain F, Kalifa C, Grill J, Dufour C: **Hypofractionated radiotherapy in the treatment of diffuse intrinsic pontine glioma in children: a single institution's experience**. *J Neurooncol* 2011, **104**(3):773-777.

22. Janssens GO, Jansen MH, Lauwers SJ, Nowak PJ, Oldenburger FR, Bouffet E, Saran F, Kamphuis-van Ulzen K, van Lindert EJ, Schieving JH *et al*: **Hypofractionation vs conventional radiation therapy for newly diagnosed diffuse intrinsic pontine glioma: a matched-cohort analysis**. *Int J Radiat Oncol Biol Phys* 2013, **85**(2):315-320.

23. Wolff JE, Driever PH, Erdlenbruch B, Kortmann RD, Rutkowski S, Pietsch T, Parker C, Metz MW, Gnekow A, Kramm CM: **Intensive chemotherapy improves survival in pediatric high-grade glioma after gross total resection: results of the HIT-GBM-C protocol**. *Cancer* 2010, **116**(3):705-712.

24. Yamasaki F, Kurisu K, Kajiwara Y, Watanabe Y, Takayasu T, Akiyama Y, Saito T, Hanaya R, Sugiyama K: **Magnetic resonance spectroscopic detection of lactate is predictive of a poor prognosis in patients with diffuse intrinsic pontine glioma**. *Neuro Oncol* 2011, **13**(7):791-801.

25. Steffen-Smith EA, Baker EH, Venzon D, Shandilya S, Bent RS, Warren KE: **Measurements of the pons as a biomarker of progression for pediatric DIPG**. *J Neurooncol* 2014, **116**(1):127-133.

26. Puget S, Beccaria K, Blauwblomme T, Roujeau T, James S, Grill J, Zerah M, Varlet P, Sainte-Rose C: **Biopsy in a series of 130 pediatric diffuse intrinsic Pontine gliomas**. *Child's Nerv Syst* 2015, **31**(10):1773-1780.

27. Bradley KA, Zhou T, McNall-Knapp RY, Jakacki RI, Levy AS, Vezina G, Pollack IF: **Motexafin-gadolinium and involved field radiation therapy for intrinsic pontine glioma of childhood: a children's oncology group phase 2 study**. *Int J Radiat Oncol Biol Phys* 2013, **85**(1):e55-60.

28. Mauffrey C: **Paediatric brainstem gliomas: prognostic factors and management**. *J Clin Neurosci* 2006, **13**(4):431-437.

29. Kebudi R, Cakir FB: **Management of diffuse pontine gliomas in children: Recent developments**. *Pediatr Drugs* 2013, **15**(5):351-362.

30. Broniscer A, Baker JN, Tagen M, Onar-Thomas A, Gilbertson RJ, Davidoff AM, Pai Panandiker AS, Leung W, Chin TK, Stewart CF *et al*: **Phase I study of vandetanib during and after radiotherapy in children with diffuse intrinsic pontine glioma**. *Journal of clinical oncology : official journal of the American Society of Clinical Oncology* 2010, **28**(31):4762-4768.

31. Broniscer A, Baker SD, Wetmore C, Pai Panandiker AS, Huang J, Davidoff AM, Onar-Thomas A, Panetta JC, Chin TK, Merchant TE *et al*: **Phase I trial, pharmacokinetics, and pharmacodynamics of vandetanib and dasatinib in children with newly diagnosed diffuse intrinsic pontine glioma**. *Clinical cancer research : an official journal of the American Association for Cancer Research* 2013, **19**(11):3050-3058.

32. Haas-Kogan DA, Banerjee A, Poussaint TY, Kocak M, Prados MD, Geyer JR, Fouladi M, Broniscer A, Minturn JE, Pollack IF *et al*: **Phase II trial of tipifarnib and radiation in children with newly diagnosed diffuse intrinsic pontine gliomas**. *Neuro Oncol* 2011, **13**(3):298-306.

33. Hipp SJ, Steffen-Smith E, Hammoud D, Shih JH, Bent R, Warren KE: **Predicting outcome of children with diffuse intrinsic pontine gliomas using multiparametric imaging**. *Neuro Oncol* 2011, **13**(8):904-909.

34. Jalali R, Raut N, Arora B, Gupta T, Dutta D, Munshi A, Sarin R, Kurkure P: **Prospective evaluation of radiotherapy with concurrent and adjuvant temozolomide in children with newly diagnosed diffuse intrinsic pontine glioma**. *Int J Radiat Oncol Biol Phys* 2010, **77**(1):113-118.

35. Porkholm M, Valanne L, Lonnqvist T, Holm S, Lannering B, Riikonen P, Wojcik D, Sehested A, Clausen N, Harila-Saari A *et al*: **Radiation therapy and concurrent topotecan followed by maintenance triple anti-angiogenic therapy with thalidomide, etoposide, and celecoxib for pediatric diffuse intrinsic pontine glioma**. *Pediatr Blood Cancer* 2014, **61**(9):1603-1609.

36. Broniscer A, Leite CC, Lanchote VL, Machado TM, Cristofani LM: **Radiation therapy and high-dose tamoxifen in the treatment of patients with diffuse brainstem gliomas: results of a Brazilian cooperative study. Brainstem Glioma Cooperative Group**. *Journal of clinical oncology : official journal of the American Society of Clinical Oncology* 2000, **18**(6):1246-1253.

37. Chassot A, Canale S, Varlet P, Puget S, Roujeau T, Negretti L, Dhermain F, Rialland X, Raquin MA, Grill J *et al*: **Radiotherapy with concurrent and adjuvant temozolomide in children with newly diagnosed diffuse intrinsic pontine glioma**. *J Neurooncol* 2012, **106**(2):399-407.

38. Broniscer A, Iacono L, Chintagumpala M, Fouladi M, Wallace D, Bowers DC, Stewart C, Krasin MJ, Gajjar A: **Role of temozolomide after radiotherapy for newly diagnosed diffuse brainstem glioma in children: results of a multiinstitutional study (SJHG-98)**. *Cancer* 2005, **103**(1):133-139.

39. Chiang KL, Chang KP, Lee YY, Huang PI, Hsu TR, Chen YW, Chang FC, Wong TT: **Role of temozolomide in the treatment of newly diagnosed diffuse brainstem glioma in children: experience at a single institution**. *Childs Nerv Syst* 2010, **26**(8):1035-1041.

40. Lesniak MS, Klem JM, Weingart J, Carson BS, Sr.: **Surgical outcome following resection of contrast-enhanced pediatric brainstem gliomas**. *Pediatr Neurosurg* 2003, **39**(6):314-322.

41. Cohen KJ, Heideman RL, Zhou T, Holmes EJ, Lavey RS, Bouffet E, Pollack IF: **Temozolomide in the treatment of children with newly diagnosed diffuse intrinsic pontine gliomas: a report from the Children's Oncology Group**. *Neuro Oncol* 2011, **13**(4):410-416.

42. Michalski A, Bouffet E, Taylor RE, Hargrave D, Walker D, Picton S, Robinson K, Pizer B, Bujkiewicz S: **The addition of high-dose tamoxifen to standard radiotherapy does not improve the survival of patients with diffuse intrinsic pontine glioma**. *J Neurooncol* 2010, **100**(1):81-88.

43. Mandell LR, Kadota R, Freeman C, Douglass EC, Fontanesi J, Cohen ME, Kovnar E, Burger P, Sanford RA, Kepner J *et al*: **There is no role for hyperfractionated radiotherapy in the management of children with newly diagnosed diffuse intrinsic brainstem tumors: results of a Pediatric Oncology Group phase III trial comparing conventional vs. hyperfractionated radiotherapy**. *Int J Radiat Oncol Biol Phys* 1999, **43**(5):959-964.

44. Bernier-Chastagner V, Grill J, Doz F, Bracard S, Gentet JC, Marie-Cardine A, Luporsi E, Margueritte G, Lejars O, Laithier V *et al*: **Topotecan as a radiosensitizer in the treatment of children with malignant diffuse brainstem gliomas: results of a French Society of Paediatric Oncology Phase II Study**. *Cancer* 2005, **104**(12):2792-2797.

45. de Aquino Gorayeb MM, Aisen S, Nadalin W, Panico Gorayeb R, de Andrade Carvalho H: **Treatment of childhood diffuse brain stem tumors: comparison of results in different treatment modalities**. *Clin Transl Oncol* 2006, **8**(1):45-49.

46. Kornreich L, Schwarz M, Karmazyn B, Cohen IJ, Shuper A, Michovitz S, Yaniv I, Fenig E, Horev G: **Role of MRI in the management of children with diffuse pontine tumors: a study of 15 patients and review of the literature**. *Pediatr Radiol* 2005, **35**(9):872-879.

47. Packer RJ, Prados M, Phillips P, Nicholson HS, Boyett JM, Goldwein J, Rorke LB, Needle MN, Sutton L, Zimmerman RA *et al*: **Treatment of children with newly diagnosed brain stem gliomas with intravenous recombinant beta-interferon and hyperfractionated radiation therapy: a childrens cancer group phase I/II study**. *Cancer* 1996, **77**(10):2150-2156.

48. Rosenfeld A, Etzl M, Bandy D, Carpenteri D, Gieseking A, Dvorchik I, Kaplan A: **Use of positron emission tomography in the evaluation of diffuse intrinsic brainstem gliomas in children**. *J Pediatr Hematol Oncol* 2011, **33**(5):369-373.

49. Doz F, Neuenschwander S, Bouffet E, Gentet JC, Schneider P, Kalifa C, Mechinaud F, Chastagner P, De Lumley L, Sariban E *et al*: **Carboplatin before and during radiation therapy for the treatment of malignant brain stem tumours: a study by the Societe Francaise d'Oncologie Pediatrique**. *European journal of cancer (Oxford, England : 1990)* 2002, **38**(6):815-819.

50. Panigrahy A, Nelson MD, Jr., Finlay JL, Sposto R, Krieger MD, Gilles FH, Bluml S: **Metabolism of diffuse intrinsic brainstem gliomas in children**. *Neuro Oncol* 2008, **10**(1):32-44.

51. Allen J, Siffert J, Donahue B, Nirenberg A, Jakacki R, Robertson P, DaRosso R, Thoron L, Rosovsky M, Pinto R: **A phase I/II study of carboplatin combined with hyperfractionated radiotherapy for brainstem gliomas**. *Cancer* 1999, **86**(6):1064-1069.

52. Khuong-Quang DA, Buczkowicz P, Rakopoulos P, Liu XY, Fontebasso AM, Bouffet E, Bartels U, Albrecht S, Schwartzentruber J, Letourneau L *et al*: **K27M mutation in histone H3.3 defines clinically and biologically distinct subgroups of pediatric diffuse intrinsic pontine gliomas**. *Acta Neuropathol* 2012, **124**(3):439-447.

53. Frappaz D, Schell M, Thiesse P, Marec-Berard P, Mottolese C, Perol D, Bergeron C, Philip T, Ricci AC, Galand-Desme S *et al*: **Preradiation chemotherapy may improve survival in pediatric diffuse intrinsic brainstem gliomas: final results of BSG 98 prospective trial**. *Neuro Oncol* 2008, **10**(4):599-607.

54. Packer RJ, Krailo M, Mehta M, Warren K, Allen J, Jakacki R, Villablanca JG, Chiba A, Reaman G: **A Phase I study of concurrent RMP-7 and carboplatin with radiation therapy for children with newly diagnosed brainstem gliomas**. *Cancer* 2005, **104**(9):1968-1974.

55. Moghrabi A, Kerby T, Tien RD, Friedman HS: **Prognostic value of contrast-enhanced magnetic resonance imaging in brainstem gliomas**. *Pediatr Neurosurg* 1995, **23**(6):293-298.

56. Sanghavi SN, Needle MN, Krailo MD, Geyer JR, Ater J, Mehta MP: **A phase I study of topotecan as a radiosensitizer for brainstem glioma of childhood: first report of the Children's Cancer Group-0952**. *Neuro Oncol* 2003, **5**(1):8-13.

57. Marcus KJ, Dutton SC, Barnes P, Coleman CN, Pomeroy SL, Goumnerova L, Billett AL, Kieran M, Tarbell NJ: **A phase I trial of etanidazole and hyperfractionated radiotherapy in children with diffuse brainstem glioma**. *Int J Radiat Oncol Biol Phys* 2003, **55**(5):1182-1185.

58. Bradley KA, Pollack IF, Reid JM, Adamson PC, Ames MM, Vezina G, Blaney S, Ivy P, Zhou T, Krailo M *et al*: **Motexafin gadolinium and involved field radiation therapy for intrinsic pontine glioma of childhood: a Children's Oncology Group phase I study**. *Neuro Oncol* 2008, **10**(5):752-758.

59. Jennings MT, Sposto R, Boyett JM, Vezina LG, Holmes E, Berger MS, Bruggers CS, Bruner JM, Chan KW, Dusenbery KE *et al*: **Preradiation chemotherapy in primary high-risk brainstem tumors: phase II study CCG-9941 of the Children's Cancer Group**. *J Clin Oncol* 2002, **20**(16):3431-3437.

60. Pirotte BJ, Lubansu A, Massager N, Wikler D, Goldman S, Levivier M: **Results of positron emission tomography guidance and reassessment of the utility of and indications for stereotactic biopsy in children with infiltrative brainstem tumors**. *J Neurosurg* 2007, **107**(5 Suppl):392-399.

61. Turner CD, Chi S, Marcus KJ, MacDonald T, Packer RJ, Poussaint TY, Vajapeyam S, Ullrich N, Goumnerova LC, Scott RM *et al*: **Phase II study of thalidomide and radiation in children with newly diagnosed brain stem gliomas and glioblastoma multiforme**. *J Neurooncol* 2007, **82**(1):95-101.

62. Wang ZJ, Rao L, Bhambhani K, Miller K, Poulik J, Altinok D, Sood S: **Diffuse intrinsic pontine glioma biopsy: a single institution experience**. *Pediatr Blood Cancer* 2015, **62**(1):163-165.

63. Zaghloul MS, Eldebawy E, Ahmed S, Mousa AG, Amin A, Refaat A, Zaky I, Elkhateeb N, Sabry M: **Hypofractionated conformal radiotherapy for pediatric diffuse intrinsic pontine glioma (DIPG): a randomized controlled trial**. *Radiother Oncol* 2014, **111**(1):35-40.

64. Hummel TR, Salloum R, Drissi R, Kumar S, Sobo M, Goldman S, Pai A, Leach J, Lane A, Pruitt D *et al*: **A pilot study of bevacizumab-based therapy in patients with newly diagnosed high-grade gliomas and diffuse intrinsic pontine gliomas**. *J Neurooncol* 2016, **127**(1):53-61.

65. Bouffet E, Raquin M, Doz F, Gentet JC, Rodary C, Demeocq F, Chastagner P, Lutz P, Hartmann O, Kalifa C: **Radiotherapy followed by high dose busulfan and thiotepa: a prospective assessment of high dose chemotherapy in children with diffuse pontine gliomas**. *Cancer* 2000, **88**(3):685-692.
